# Supplementary material for: Intestinal Shedding of SARS-CoV-2 in Children: No Evidence for Infectious Potential
Source: Microorganisms. 2022 Dec 22;11(1):33. doi: 10.3390/microorganisms11010033 (PMC9864026; doi:10.3390/microorganisms11010033)
Supplement: Supplementary file 1 [file microorganisms-11-00033-s001.zip › microorganisms-2103998-SI.pdf]

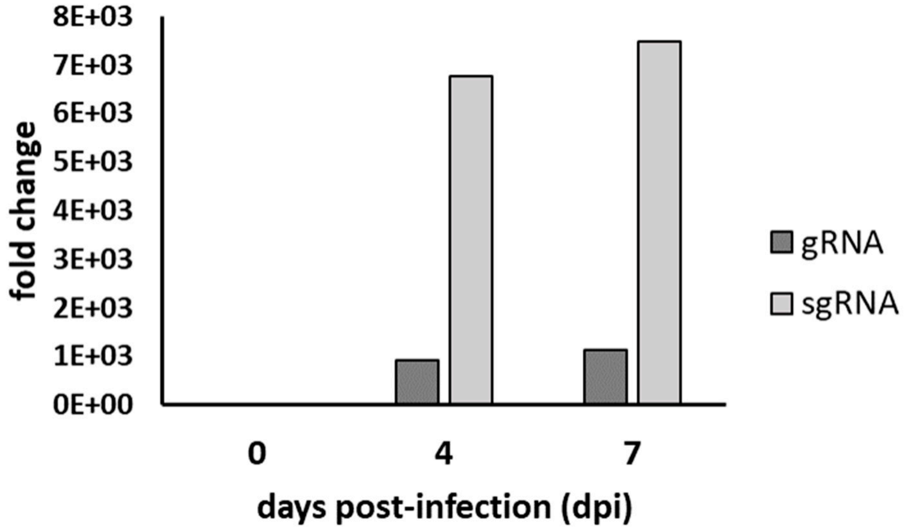

Figure S1. Positive controls used for SARS-CoV-2 gRNA and sgRNA detection in infected stool samples. Quantification of gRNA and sgRNA in specimens taken at 0, 4 and 7 dpi from Vero-E6 cell culture incubated with SARS-CoV-2 derived from stool specimens. The viral loads are indicated as fold change normalized to the 0 dpi sample.
